# The Acute Immune Response in Sheep Following Immunization with *Toxoplasma gondii* Tachyzoites or Parasite-Derived Glycoconjugates

**DOI:** 10.3390/vetsci12100928

**Published:** 2025-09-24

**Authors:** Patrícia Oliveira Meira-Santos, Gabriela Cruz Piedade, Maria Tereza Guedes, Dan Loureiro, José Tadeu Raynal, Roberto Meyer, Letícia Vicentini, Luiz Soares, Blima Fux, Ricardo Wagner Portela

**Affiliations:** 1 Veterinary Medicine Department, Universidade Federal de Sergipe, São Cristóvão 49107-230, SE, Brazil; patriciameira@academico.ufs.br; 2 Laboratory of Immunology and Molecular Biology, Health Sciences Institute, Universidade Federal da Bahia, Salvador 40110-100, BA, Brazil; 3 Tropical Medicine Unit, Health Sciences Center, Universidade Federal do Espírito Santo, Vitória 29047-105, ES, Brazil

**Keywords:** toxoplasmosis, glycobiology, small ruminants, veterinary parasitology

## Abstract

Toxoplasmosis is a relevant parasitic infection in sheep, with ovine meat an important source of human exposure. Accurate detection of the early immune response to *Toxoplasma gondii* is essential for preventing reproductive losses and improving diagnostic strategies. This study evaluated the kinetics of the acute immune response in eighteen sheep experimentally exposed to live tachyzoites or immunized with parasite-derived glycoconjugates (GlyC). Animals were divided into three groups and injected with saline solution, tachyzoites, or glycoconjugates combined with an adjuvant. Infected sheep developed specific IgM antibodies against both lysate and glycoconjugate antigens from day 4, and IgG against glycoconjugates from day 12 post-infection. Glycoconjugate-immunized sheep produced IgM against lysate antigens from day 4, and IgG against both antigens from day 12. Flow cytometry revealed a significant increase in circulating CD8+ T cells and a reduction in MHC class II+ cells on day 60 in the infected group. These findings demonstrate the early humoral and cellular immune response profiles following infection or GlyC immunization. This supports their future application in diagnostic tests or as vaccine candidates against toxoplasmosis in sheep.

## Simple Summary

Toxoplasmosis is a parasitic disease that affects both animals and humans, posing a particular risk to sheep by causing reproductive losses and serving as a source of infection for people through contaminated meat. In this study, we evaluated how the sheep’s immune system responds after exposure to live parasites or glycoconjugated molecules extracted from the parasite. The objective was to determine whether these purified molecules could stimulate immune responses similar to those triggered by the infection. Sheep were assigned to three groups: one received a saline solution (control), another was infected with live parasites, and the third was immunized with the parasite-derived molecules. Over a period of 60 days, we monitored the production of antibodies and the behavior of immune cells in all groups. Both infection and immunization activated the immune system, with the immunized group showing significant responses even without previous exposure to the live parasite. These findings suggest that parasite-derived glycoconjugated molecules may be useful for developing safer diagnostic tools or vaccines for toxoplasmosis in livestock, helping to prevent economic losses in animal production and reduce the risk of transmission to humans.

## 1. Introduction

The phylum Apicomplexa comprises obligate intracellular parasites, including *Toxoplasma gondii*, a causative agent of significant medical and veterinary diseases. Infection by *T. gondii* in sheep is associated with abortions, stillbirths, and congenital diseases, resulting in substantial impacts on animal health and economic losses in sheep farming worldwide [1]. In Brazil, the prevalence of *T. gondii* infection in sheep ranges from 7% to 85%, indicating widespread parasite circulation [2].

In sheep, infection occurs through ingestion of oocysts or transplacental transmission, followed by the formation of tissue cysts containing bradyzoites, which maintain the infectious cycle [3]. The *T. gondii* infection is usually asymptomatic in non-pregnant ruminants; some animals can present nonspecific symptoms, such as fever, apathy, anorexia, diarrhea, appetite loss, nasal discharge, and coughing for a few days [4]. Pregnant mammals that become prime-infected during pregnancy can present an immunomodulatory response, which favors the vertical transmission of the parasite; this situation expresses the fact that the response to *T. gondii* can vary depending on the animal’s health history or infectious status [5].

The kinetics of the immune responses against the parasite in sheep are still poorly understood. IgM is the first antibody to be produced against the parasite, and can be detected in the early stages of the infection with *T. gondii*, being considered an acute phase marker of toxoplasmosis. Parasite-specific IgG is produced soon after IgM, and its production can last longer than IgM, being in this way considered a marker of the chronic phase of the infection. However, it is noteworthy that there are individual variations in detecting these immunoglobulins, since sheep experimentally infected with the parasite may not present detectable levels of IgM and IgG [6].

The cellular immune response against *T. gondii* is correlated with a protective immune profile. It is mediated by the interaction of Th1, Th2, and Treg cells and their cytokines (IFN-γ and TNF, IL4, and IL10, respectively), which can generate either pro- or anti-inflammatory responses depending on the stimulus [7]. Specifically, MHC I+ and MHC II+ cells can present *T. gondii*-derived epitopes to CD8+ and CD4+ T cells, respectively, mediating the activation of these lymphocytes; CD8+ cells are responsible for the killing of parasite-infected cells with a consequent inhibition of its proliferation, and CD4+ cells produce cytokines that orchestrate the acquired immune response to the protozoan [8].

The immunodiagnosis of the *T. gondii* infection is a matter of discussion among researchers. Indirect immunofluorescence techniques using whole tachyzoites and immunoenzymatic assays employing total lysate antigen have been used to detect parasite-specific antibodies in humans and animals. Still, they may present problems with sensitivity and specificity [9]. In this way, recombinant proteins are being proposed as molecules that can confer higher levels of specificity [10]; in addition, it was already described that the use of parasite-derived glycoconjugates (GlyC) in ELISA resulted in enhanced levels of sensitivity, especially for the detection of specific IgM in the acute phase of toxoplasmosis [11].

Glycosylphosphatidylinositol (GPI), a molecule characterized by a conserved core structure of phosphatidylinositol–lipid linked to a glycan consisting of non-acetylated glucosamine and mannose residues [12], is essential for the survival of *T. gondii*, and GPI-anchored proteins have been implicated in host cell attachment and modulation of the immune response [13]. GPIs from various protozoa are recognized by toll-like receptors, leading to the production of tumor necrosis factor (TNF), interleukin-12 (IL-12), and nitric oxide by macrophages [14]. *T. gondii* tachyzoites-derived GPIs stimulate inflammatory responses by inducing macrophage TNF production through NF-kB activation [15]. In addition, only *T. gondii* GPIs bearing the unique glucose-N-acetylgalactosamine side branch are immunogenic, and this structure is widely distributed among *T. gondii* isolates [12]. Given that GPI-anchored proteins are abundant on the surface of *T. gondii* and play a crucial role in immunomodulation [16], they represent promising targets for immunological studies.

Therefore, this study aimed to analyze the kinetics of the acute humoral and cellular immune responses in sheep infected with *T. gondii* tachyzoites or immunized with parasite-derived GPI-anchored proteins, intending to contribute to the development of more effective diagnostic and vaccine strategies.

## 2. Materials and Methods

### 2.1. Animals

Thirty male and female sheep were selected through clinical examination and transported to an experimental farm. Further clinical evaluations (rectal temperature, body score, occurrence of lymphadenomegaly, mucosal integrity and color, occurrence of nasal or ocular discharges, skin turgor), fecal examinations (presence of gastrointestinal parasites’ eggs in feces), and indirect immunofluorescence assays (IFAT) for the detection of *Toxoplasma gondii* and *Neospora caninum*–specific antibodies, were conducted to confirm health status and absence of prior exposure.

Fourteen animals were excluded due to the presence of anti-*T. gondii* IgG, indicating previous infection. The remaining sixteen animals received preventive anti-helminthic treatment and underwent additional clinical examinations before the start of the experiments. Throughout the study, animals were maintained under semi-intensive management with daily access to mineral salt, forage, and water ad libitum.

### 2.2. Parasites

*T. gondii* RH strain tachyzoites were maintained both in vivo and in vitro. Female Swiss mice were inoculated intraperitoneally with 10^6^ tachyzoites suspended in saline. Five days post-inoculation, mice were euthanized, and parasites were harvested from the peritoneal fluid using saline solution. These tachyzoites were used for the in vitro culture.

VERO kidney epithelial cells were maintained at 37 °C with 5% CO_2_ in RPMI medium (Sigma Aldrich, Saint Louis, MO, USA) supplemented with antibiotics (5 mg/mL penicillin, 5 mg/mL streptomycin, 10 mg/mL neomycin; Gibco, Waltham, MA, USA) and 10% horse serum (Gibco). VERO cells were cultured until reaching approximately 90% confluence. Subsequently, the tachyzoites that were recovered in vivo were added to the cultures following medium replacement. Parasite recovery was performed after three days by washing the cultures three times with sterile physiological solution, followed by centrifugation at 2500 rpm for ten minutes. These parasites were used for the experimental animals’ infection and for obtaining GlyC. Also, a pellet containing 10^10^ tachyzoites was sonicated on ice five times for 30 s each and resuspended in saline solution to produce *T. gondii* lysate antigen (LA); the LA was obtained to conduct immunoenzymatic assays (ELISAs). Protein concentration was quantified using the Micro BCA Protein Assay Kit (Thermo Scientific, Waltham MA, USA).

### 2.3. Glycoconjugate Extraction

Glycoconjugates derived from *T. gondii* were purified following the method described by Portela et al. [16]. Briefly, the 10^10^ tachyzoite pellet was subjected to partitioning with a chloroform–methanol–water solution (5:10:4) for 1 h at room temperature, followed by centrifugation at 5000× *g* for 15 min at 10 °C. This step was repeated twice on the resulting pellet. Subsequently, the pellets were partitioned with 9% butan-1-ol in water and centrifuged as described above for three consecutive cycles. The resulting supernatants were pooled and concentrated by rotary evaporation at 4 °C, 45 °C, and 85.5 kPa, generating the fraction designated F3. This fraction contains the parasite’s glycoconjugates, and protein concentrations were determined as described for lysate antigen (LA). F3 was stored at −20 °C until use.

### 2.4. Experimental Design

The sixteen remaining animals were between 8 and 18 months old. Female sheep underwent ultrasound examination to confirm the absence of pregnancy. The animals were divided into three groups: G1, saline control (*n* = 4); G2, immunized with GlyC (*n* = 5); and G3, infected with live tachyzoites (*n* = 7). G1 received 1.0 mL of sterile saline solution per animal. G2 was injected subcutaneously with 250 µg of glycoconjugates combined with 1.5 mg of saponin in 1.0 mL of saline solution, per animal. G3 was infected subcutaneously with 1 × 10^6^ *T. gondii* RH strain tachyzoites per animal in 1.0 mL of saline solution [17]. All administrations were performed in the dorsal neck region. Saponin was chosen as an adjuvant since it is an adequate adjuvant for the hydrophobic profile of the GlyC solution, and it has already been shown to be a proper adjuvant to be used in sheep [18,19].

Blood samples for humoral immune response evaluation were collected from the jugular vein using Vacutainer tubes without anticoagulant on days 0, 2, 4, 6, 8, 10, 12, 15, 20, 25, 30, 40, 50, and 60 post-inoculations (p.i.). For cellular immune response analysis, blood was collected in EDTA-containing tubes on days 0, 7, 15, 30, and 60 p.i. All procedures were conducted by veterinarians to ensure proper clinical monitoring and adherence to protocols.

### 2.5. IgM ELISA

Anti-LA and anti-GlyC IgM levels were quantified using an indirect enzyme-linked immunosorbent assay (ELISA) [11]. Antigens (10 µg/mL for LA and 6 µg/mL for GlyC) were diluted in carbonate buffer (pH 9.6) and coated onto high-binding microtiter plates (100 µL/well; Nunc, Roskilde, Denmark). Plates were incubated at 4 °C for 16 h in a humid chamber, then washed three times with phosphate-buffered saline containing 0.05% Tween 20 (PBS-T). Plates were blocked with 5% bovine serum albumin (BSA) in PBS for 2 h at 37 °C. After three washes, serum samples and standards were diluted 1:50 (LA) or 1:100 (GlyC) in PBS with 1% BSA (PBS-BSA) and added in triplicate. Plates were incubated for 1 h at 37 °C, followed by six washes with PBS-T. Anti-sheep IgM horseradish peroxidase–conjugated antibody (Bethyl Laboratories), diluted 1:10,000 in PBS-BSA, was added (100 µL/well) and incubated for 1 h at 37 °C. After six washes, substrate solution containing 4 mg/mL o-phenylenediamine (OPD, Sigma), 0.04% H_2_O_2_ in 10 mM Na_2_HPO_4_ and 100 mM citric acid (pH 5.0) was added (100 µL/well). Color development proceeded in the dark for 10 min (LA) or 15 min (GlyC), then the reaction was stopped by adding 100 µL of 1.5 M H_2_SO_4_. Optical density was measured at 492 nm using an ELISA plate reader (Multiskan, Titertek, Huntsville, AL, USA).

Positive and negative controls consisted of pools of 10 IgM-positive and 10 IgM-negative sera, respectively, taken from sheep bred in Bahia state (Brazil) and previously defined by indirect immunofluorescence assay (IFAT). All samples and controls were tested in triplicate, and mean absorbance values were calculated.

### 2.6. IgG ELISA

ELISA was employed to detect anti-LA and anti-GlyC specific IgG [11]. Antigens (2 µg/mL for LA and 10 µg/mL for GlyC) were diluted in carbonate buffer (pH 9.6) and coated onto high-binding microtiter plates (100 µL/well; Nunc). The protocol followed that of the IgM-ELISA, with modifications in blocking and diluents: nonspecific binding sites were blocked with 5% casein from cow’s milk, and serum samples and antibody conjugates were diluted in PBS containing 1% casein (PBS-M).

For ELISA with LA, serum samples and controls were diluted 1:400, and the conjugated antibody was diluted 1:20,000. For GlyC ELISA, samples and standards were diluted 1:200, and the conjugated antibody was diluted 1:10,000. Positive and negative controls comprised pools of 10 sheep serum samples previously characterized by IFAT for specific IgG. All samples and controls were tested in triplicate, and mean absorbance values were calculated.

### 2.7. Flow Cytometry

The percentages of CD4+, CD8+, and MHC class I and II positive cells were assessed by flow cytometry. Three animals from G1, four from G2, and five from G3 were selected for this analysis. Blood samples were collected in EDTA-containing vacutainer tubes on days 0, 7, 15, 30, and 60 post-injections. Aliquots of 100 µL blood were incubated with 1:50 dilutions of mouse monoclonal IgG1 antibodies against CD4 and CD8 (VMRD Inc., Pullman, WA, USA) for 15 min. Subsequently, samples were incubated in the dark for 15 min with FITC-conjugated anti-mouse IgG secondary antibody (Rockland Immunochemicals Inc., Pottstown, PA, USA) diluted 1:100. Red blood cell lysing buffer (1:10 dilution, 500 µL per tube) was added and incubated for 20 min in the dark. After washing with 1 mL PBS, samples were centrifuged at 2200× *g* for 3 min, supernatants discarded, and pellets resuspended in 500 µL 0.9% sodium chloride.

A similar protocol was used for MHC class I and II detection. Mouse anti-sheep MHC class II DQ/DR R-phycoerythrin-conjugated monoclonal antibody and mouse anti-sheep MHC class I FITC-conjugated monoclonal antibody (AbD Serotec, Kidlington, United Kingdom) were added at 2.5 µL (200 µg/mL) per tube. The subsequent steps followed those described above.

Data was acquired using a FACScalibur flow cytometer (Becton Dickinson, San Jose, CA, USA) using CellQuest software version 5.1 (Becton Dickinson, Mississauga, ON, Canada). Data analysis was conducted with FlowJo software version 10 (Tree Star Inc., Chico, CA, USA).

### 2.8. Statistical Analyses

Data were analyzed using SPSS version 12.0. The Mann–Whitney U test was applied to assess differences in specific IgG and IgM production and immune cell marker expression among experimental groups, with significance set at *p* < 0.05.

### 2.9. Ethical Aspects

This study was approved by the Ethics Committee on Animal Use in Experimentation (CEUA) of the Federal University of Ceará, Cariri Campus, under protocol number 001/2013.

## 3. Results

### 3.1. Parasite Glycoconjugate Production

The concentrations of 2.3 mg/mL and 1.8 mg/mL were achieved in the production of LA and GlyC antigens, respectively. The efficiency of GlyC extraction and purification was confirmed by SDS-PAGE (Supplementary Figure S1), and the molecular profile was the same as the one previously observed for the GlyC solution extracted using the same methodology described herein [20]. The immune recognition profile of the GlyC solution was confirmed by Western blot (Supplementary Figure S2), using serum samples with positive and negative results for *T. gondii*-specific antibody detection, as previously defined by IFAT, and also showed previously described profiles [11,16].

### 3.2. Clinical Signs and Humoral Immune Response

During the experimental period, veterinarians from the farm where the animals were allocated monitored the sheep for the occurrence of nasal discharges, apathy, diarrhea, skin lesions, and cutaneous reactions due to the inoculations. None of these clinical signs was detected.

**Figure 1:**
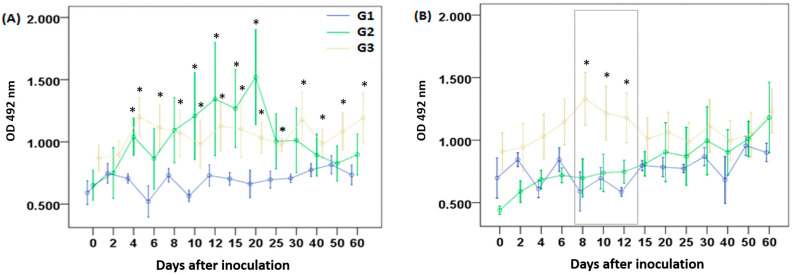
Specific IgM levels against (**A**) lysate antigen and (**B**) *T. gondii*-derived glycoconjugates. Sheep were injected once, subcutaneously, with saline solution (G1), GlyC + saponin (G2), or live tachyzoites (G3). Values are presented as means ± standard errors. Significant differences between G2 or G3 and G1 groups, determined by the Mann–Whitney test, are indicated by an asterisk (*) at *p* < 0.05. The box in (**B**) shows the period of significant differences between G3 and G1 regarding antibody levels.

On day 4 p.i., an increase in anti-LA IgM levels was observed in G2 and G3 compared to the control group. In G3, specific antibody levels remained elevated throughout the 60-day follow-up, whereas in G2, levels began to decline after day 25 (Figure 1A). A significant increase in IgM against GlyC was observed in G3, peaking on day 8, compared to the control group and G2; however, this increase was not sustained over time, and those differences disappeared after day 12 (Figure 1B).

**Figure 2:**
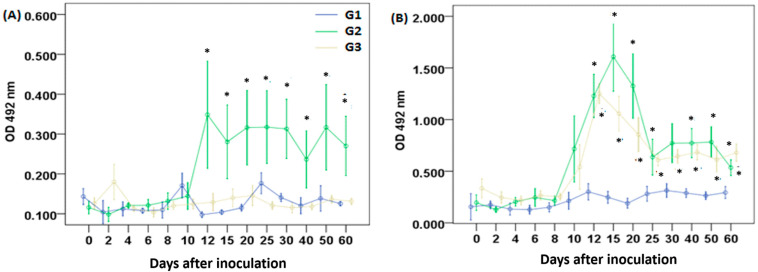
Specific IgG levels against (**A**) lysate antigen and (**B**) *T. gondii*-derived glycoconjugates. Sheep were injected once, subcutaneously, with saline solution (G1), glycoconjugates plus saponin (G2), or live tachyzoites (G3). Values are presented as means ± standard errors. The symbol “*” indicates significant differences between the control group (G1) and the treatment groups (G2 and G3) in both panels, according to the Mann–Whitney test, with *p* < 0.05.

A significant increase in IgG anti-LA levels in G2 began on day 12 p.i., remaining statistically different from G1 and G3 between days 12 and 60; G3 did not present significant production of IgG anti-LA (Figure 2A). IgG anti-GlyC levels increased in G2 and G3 starting on day 12 p.i., reaching a peak on day 15 in G2 and on day 12 in G3. Significant levels of IgG anti-GlyC were detected in both groups until the end of the experimental period (60 days p.i.) (Figure 2B).

### 3.3. Cellular Immune Response

**Figure 3:**
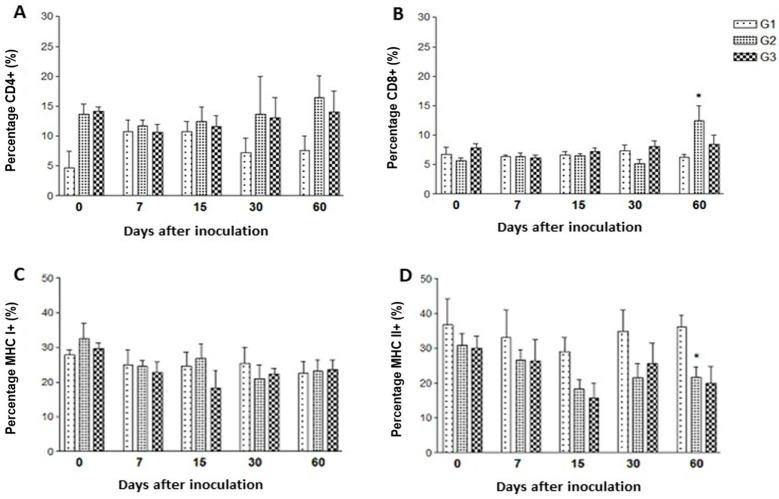
Percentages of (**A**) CD4+, (**B**) CD8+, (**C**) MHC class I+, and (**D**) MHC class II+ cells in peripheral blood of sheep inoculated with saline (control group, G1), *Toxoplasma gondii*-derived glycoconjugates (G2), or *T. gondii* RH strain tachyzoites (G3). Significant differences between groups G2 or G3 compared to G1, determined by the Mann–Whitney test, are indicated by an asterisk (*), with significance set at *p* < 0.05.

No significant statistical differences were seen between the experimental groups for the percentages of CD4+ cells (Figure 3A). On day 60 post-inoculation (p.i.), CD8+ cell counts in the GlyC-immunized group (G2) were modestly, but significantly, higher when compared to the control group (G1) (*p* = 0.034, and a 1.857-fold change) (Figure 3B). The tachyzoite-inoculated group (G3) also showed increased CD8+ counts (a 1.625-fold change compared to the control group) at day 60 p.i., but without statistical significance (Figure 3B).

Regarding MHC I+ cells, it was not possible to observe significant statistical differences between the groups during the experimental period (Figure 3C). Throughout the evaluated time points, MHC class II+ cell counts in groups G2 and G3 were consistently lower than those in the control group. However, a statistically significant difference between G1 and G2 (with a 0.52-fold change) was observed only at day 60 p.i. (Figure 3D).

## 4. Discussion

The use of glycoconjugates as vaccine antigens is becoming more explored in the scientific literature, since it was already described that they can stimulate the production of IgM, IgG [11], and IgA [16], and they can also stimulate the cellular immune response when linked to proteins, being considered as promising new adjuvants [21]. As *T. gondii* is covered by GPI-anchored proteins [22], and toxoplasmosis is a disease of significant importance for small ruminants, we investigated the immune stimulation induced by a GPI-anchored protein-enriched solution in sheep. As described below, a single immunization with GlyC derived from *T. gondii* tachyzoites induced an immune response in these animals.

The animals injected with 250 µg of GlyC (G2) produced detectable levels of IgM and IgG antibodies against LA. The presence of IgM antibodies against whole-tachyzoite antigens is indicative of recent infection and is commonly used in diagnostic tests to identify acute toxoplasmosis. Some studies have evaluated the presence of IgM antibodies against lysate antigen or recombinant multiepitope peptides (rMEP) in humans. However, they did not analyze the kinetics of the response due to the difficulty in determining the time of infection [23].

GPIs are relevant molecules to all eukaryotes, playing a key role in the *T. gondii* infectious process and in the immune response against the parasite, since they are essential targets of IgM antibodies [16,22]. A protein known as SAG1 (or p30) accounts for almost 5% of all *T. gondii* antigens, being a GPI-anchored, immunodominant, and stage-specific antigen present in the tachyzoite stage but absent in the bradyzoite stage. It is one of the first antigens recognized by IgM antibodies in humans and mice during recent toxoplasmosis, although specific SAG1 antibodies can also be found during chronic infection [24]. Additionally, it has been observed that the primary targets of IgM antibodies against *T. gondii* in humans are glycoinositolphospholipids (GIPLs), which are free GPI-anchors without proteins [11]. These authors developed an IgM ELISA using purified GIPLs as antigens, achieving 95.5% specificity and 81.8% sensitivity. They also found that IgG antibodies preferentially target GPI-anchored proteins, making them helpful in discriminating between infected and uninfected individuals. This situation can explain the lack of significant anti-GlyC IgM antibody production in sheep immunized with the GPI-anchored enriched solution, since GIPLs are present in LA but not in the GlyC solution used herein [16,20]; also, it must be considered that the GPI anchor is highly hydrophobic, and can make hydrophobic interactions with the ELISA plates, exposing the proteins, and with a consequent masking of the GPI motif and lack of binding with the serum specific IgM antibodies.

Although specific IgM antibodies are the main serological markers for diagnosing acute toxoplasmosis, their levels can persist for several months post-infection [25]. In animals, few studies have evaluated the IgM response during the acute phase of *T. gondii* infection in sheep or following vaccination [26,27]. A previous work assessing DNA immunization with genes encoding *T. gondii* antigens in sheep focused on humoral immune response by measuring IgG production [28]. Therefore, our study is important when evaluating the kinetics of IgM production following immunization.

Immunization of sheep with GlyC induced significant production of IgG antibodies against both LA and GlyC. IgG antibodies against GlyC were also detected in animals inoculated with *T. gondii* tachyzoites. A weak specific IgG peak was found in sheep immunized with *T. gondii*-derived SAG1 and SAG2 proteins (both GPI-anchored) 12 days after inoculation, in a similar way to what was found in our study [29]. Corroborating our results, but in an experiment conducted in mice, SAG1 and SAG3 (another GPI-anchored protein) induced specific IgG detectable levels from 15 days after inoculation [30]. However, it is noteworthy that it was not possible to detect significant anti-LA IgG levels in sheep infected with live tachyzoites, while anti-GlyC IgG antibodies were significantly produced by those animals. This situation may be related to a limitation of sensitivity when using LA as an antigen in immunodiagnostic assays [31], to the inoculum dosage and the route of infection, or even to the fact that Group 3 received a GPI-anchored protein-enriched solution, which can lead to an enhanced immunologic stimulation. In addition, our results confirm the fact that GlyC-based immunodiagnostic assays have an improved sensitivity in the detection of anti-*T. gondii* IgG, as already described for humans [11].

IgG antibodies against GlyC have also been reported in humans, with increased production of IgG subclasses associated with Th1 cellular responses (IgG1 and IgG3) [16]. Although IgG subclasses were not analyzed in this study, it is possible that the IgG response induced by infection or immunization with GlyC is a consequence of the activation of the Th1 profile, similar to that observed in humans. However, it is noteworthy that the presence of *T. gondii*-specific antibodies alone is not correlated with protection, being mainly considered as a marker of infection [32].

In immunocompetent hosts, antibodies contribute to immunity, but control of *T. gondii* infection is predominantly mediated by cellular immune responses. CD8+ T cells act as effector cells, while CD4+ T cells play a central role in regulating immunity against *T. gondii* [33]. Protective immunity against *T. gondii* typically involves a Th1-type response, characterized by IFN-γ production by effector T cells; however, excessive production of pro-inflammatory cytokines can cause tissue damage, leading to the release of anti-inflammatory cytokines that may promote parasite proliferation [34]. Also, during the acute phase of toxoplasmosis, CD4+ T cells expand less efficiently than CD8+ T cells [35], and infected CD4+ T cells undergo rapid apoptosis through mechanisms such as caspase inhibition and with a consequent lack of antigenic stimulation, resulting in reduced responsiveness and favoring persistence of the protozoan [36,37]. This mechanism may explain the absence of significant changes in circulating CD4+ T cell percentages observed in our study.

Our experiment demonstrated a significant increase in CD8+ T cells in peripheral blood 60 days after GlyC injection, although no changes in MHC class I+ cells were detected. Previous studies indicate that direct antigen presentation by infected cells in the host tissues activates CD8+ T cells [38]. Indeed, activated dendritic cells can cross-present antigens to CD8+ T cells in lymph nodes [39]. The absence of increased MHC class I+ cells in our study may be related to these specific activation mechanisms at those tissues, since we screened blood cells.

MHC presentation plays a critical role in toxoplasmosis resolution. In mice, the MHC class I allele d efficiently presents GRA6, a key T cell epitope [40]. In humans, the influence of MHC is more subtle; for example, increased susceptibility to congenital infection has been associated with the DQ3 haplotype of MHC class II. Unlike murine models, MHC class I genes do not appear to correlate with human susceptibility [41]. Larger studies involving different sheep breeds and crossbreeds, preferably including MHC polymorphism analysis, are necessary to determine whether these molecules have a similar role in sheep.

Sixty days after GlyC inoculation, a statistically significant decrease in MHC class II+ cells was observed in peripheral blood. It has been demonstrated that *T. gondii* inhibits MHC class II synthesis in macrophages pre-activated with IFN-γ, affecting both infected and neighboring uninfected cells, independently of IL-10, TGF-β, nitric oxide, or prostaglandin E2 synthesis [42]. This inhibition has also been reported in various cell types from mice, rats, and humans, including immature dendritic cells, potentially delaying lymphocyte activation [43].

In mice, vaccination with recombinant adenovirus encoding the circumsporozoite protein (CS) of *Plasmodium yoelii* lacking the GPI-anchoring motif elicited a Th1 protective immunity. The CS protein without GPI showed more uniform intracellular distribution and was preferentially processed by cytosolic proteases, indicating that the GPI anchor may impair immune responses in vivo. Also, a preferential production of IgG2 and IFN-γ secretion by CD8+ T lymphocytes was reported in mice immunized with CS with partial or no GPI anchoring. Although GPI did not affect CS expression or secretion, its deletion enhanced B and T cell responses by altering CS intracellular distribution and processing [44]. In *Schistosoma mansoni*, cleavage of the GPI anchor followed by immunization with the anchorless protein induced a mixed immune response, increasing IgG, IgG1, IgG2, and IgE levels 30 days post-administration. IFN-γ, TNF, and IL-10 production were also elevated seven days after the second booster, with vaccinated mice showing partial protection against parasite challenge and reduced liver pathology [45]. In our study, the significant humoral response induced suggests that the GPI anchors present in the GlyC-enriched preparation did not impair the immune activation.

In summary, GlyC derived from *T. gondii* effectively induced humoral immunity, with high levels of IgG production and increased CD8+ T cell numbers in peripheral blood. These findings support the immunogenicity of *T. gondii*-derived GlyC in sheep and suggest their potential use as diagnostic markers for acute toxoplasmosis as well as components in vaccine development.

## Data Availability

The original contributions presented in this study are included in the article/Supplementary Material. Further inquiries can be directed to the corresponding authors.

## Supplementary Materials

The following supporting information can be downloaded at: https://www.mdpi.com/article/10.3390/vetsci12100928/s1, Figure S1: Molecular profile of the GPI-anchored protein-enriched solution used in this study; Figure S2: Recognition of the GPI-anchored proteins from the glycoconjugate solution used in this study.

## Abbreviations

BSA	Bovine Serum Albumin
CD4+	Cluster of Differentiation 4 positive cells
CD8+	Cluster of Differentiation 8 positive cells
CEUA	Ethics Committee on Animal Use in Experimentation
CS	Circumsporozoite Protein
EDTA	Ethylenediaminetetraacetic Acid
ELISA	Enzyme-Linked Immunosorbent Assay
F3	Fraction 3 (from glycoconjugate extraction process)
FITC	Fluorescein Isothiocyanate
GIPLs	Glycosylinositolphospholipids
GlyC	Glycoconjugates
GPI	Glycosylphosphatidylinositol
GRA6	Dense Granule Antigen 6
IFAT	Indirect Fluorescent Antibody Test
IFN-γ	Interferon-gamma
Ig	Immunoglobulin E
IL	Interleukin
LA	Lysate Antigen
MAG1	Matrix Antigen 1
MHC	Major Histocompatibility Complex
NK cells	Natural Killer Cells
OPD	o-Phenylenediamine
p.i.	Post-inoculation
PBS	Phosphate-Buffered Saline
rpm	Revolutions per Minute
SAG1	Surface Antigen 1
SPSS	Statistical Package for the Social Sciences
Th1	T helper type 1
TNF	Tumor Necrosis Factor
