# Supplementary material for: The Acute Immune Response in Sheep Following Immunization with Toxoplasma gondii Tachyzoites or Parasite-Derived Glycoconjugates
Source: Vet Sci. 2025 Sep 24;12(10):928. doi: 10.3390/vetsci12100928 (PMC12567891; doi:10.3390/vetsci12100928)

**Supplementary Figure 1. Molecular profile of the GPI-anchored protein-enriched solution used in this study.** After the purification, the solution containing the glycoconjugates was applied to a sodium dodecyl sulfate-polyacrylamide gel electrophoresis (SDS-PAGE) system, in a 12.5% acrylamide-bis-acrylamide gel, which was further stained with silver nitrate. MW - molecular weight standard; F3 – GPI-anchored protein-enriched solution. The numbers on the left represent the molecular weights of the proteins in the standard, in kDa.

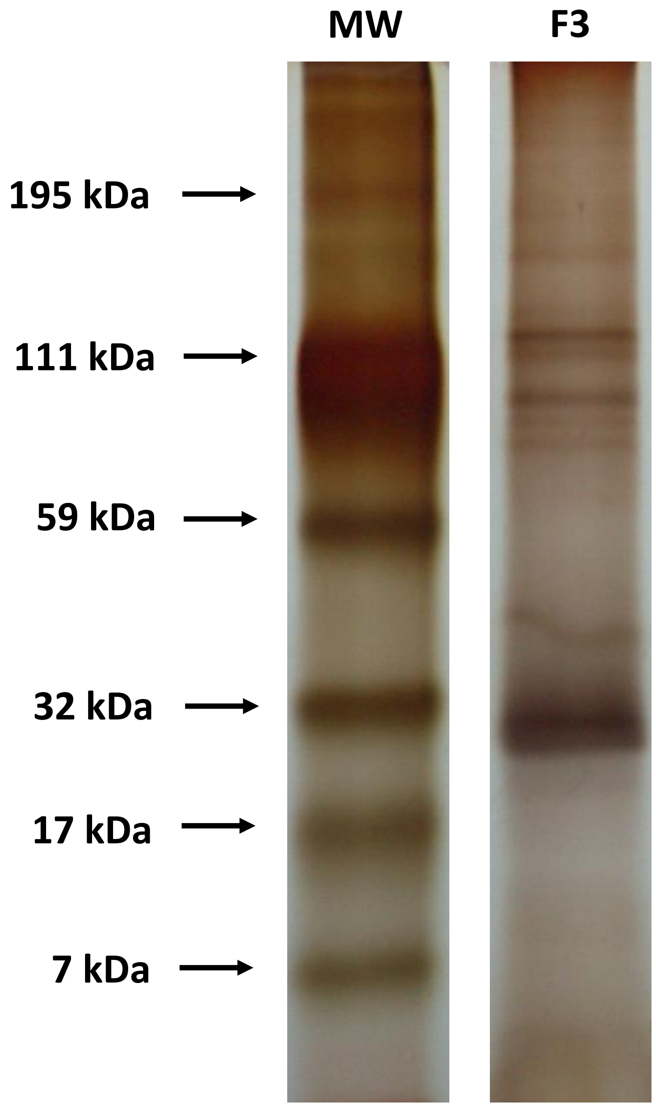

Supplement: Supplementary file 1 [file vetsci-12-00928-s001.zip › vetsci-3848240-Supplementary Figure S1.pdf]
