# Supplementary material for: The Acute Immune Response in Sheep Following Immunization with Toxoplasma gondii Tachyzoites or Parasite-Derived Glycoconjugates
Source: Vet Sci. 2025 Sep 24;12(10):928. doi: 10.3390/vetsci12100928 (PMC12567891; doi:10.3390/vetsci12100928)

Supplementary Figure 2. Recognition of the GPI-anchored proteins from the glycoconjugate solution used in this study. After SDS-PAGE, the proteins were transferred to nitrocellulose membranes, which were blocked and incubated with a pool of serum samples with positive (POS) or negative (NEG) results for *T. gondii*-specific antibody detection, as defined by IFAT. The numbers on the left represent the molecular weights of the proteins in the standard, in kDa.

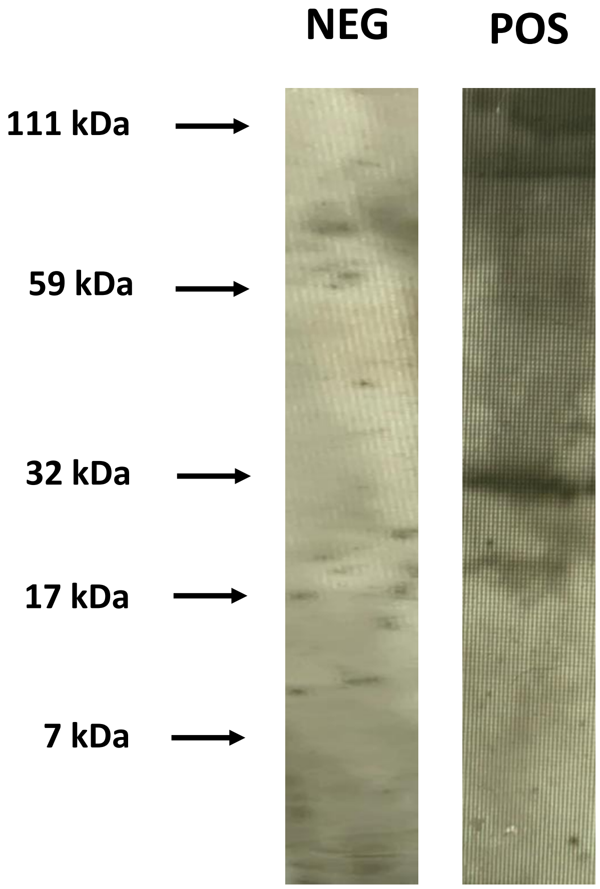

Supplement: Supplementary file 1 [file vetsci-12-00928-s001.zip › vetsci-3848240-Supplementary Figure S2.pdf]
